# ‘Where are the dead flies!’: perceptions of local communities towards the deployment of Tiny Targets to control tsetse in the Democratic Republic of the Congo

**DOI:** 10.1136/bmjgh-2021-006879

**Published:** 2022-01-06

**Authors:** Catiane Vander Kelen, Alain Mpanya, Epco Hasker, Erick Miaka, Ruth Nzuzi, Steve Torr, Dennis Perez, Justin Pulford

**Affiliations:** 1 Public Health, Institute of tropical Medicine, Antwerp, Belgium; 2 Coordination, Programme National de Lutte contre la Trypanosomiase Humaine Africaine, Kinshasa, Congo; 3 Public Health, Institute of Tropical Medicine, Antwerp, Belgium; 4 Vector Biology, Liverpool School of Tropical Medicine, Liverpool, UK; 5 Epidemiology, Pedro Kouri Institute of Tropical Medicine Center for Diagnostic and Reference Research, La Habana, Cuba; 6 International Public Health, Liverpool School of Tropical Medicine, Liverpool, UK

**Keywords:** qualitative study, human african trypanosomiasis

## Abstract

The National Programme for the control of human African trypanosomiasis in Democratic Republic of Congo includes a large-scale vector control operation using Tiny Targets. These are small panels of insecticide-impregnated cloth that are deployed in riverine habitat where tsetse flies concentrate. The effectiveness of Tiny Targets depends partly on acceptance by local communities. In 2018, we conducted research to explore the perception and acceptability of Tiny Targets in two different village clusters where Tiny Targets had been deployed by the local community or external teams. We conducted fourteen focus group discussions and seven semistructured interviews in three villages from each cluster in the Yasa Bonga health zone. Our findings showed that acceptability was better in the cluster where communities were involved in the deployment of Tiny Targets. Also in this cluster, awareness about Tiny Targets was satisfactory and the project was implemented within local customs, which promoted a positive perception of Tiny Targets and their benefits. In the cluster where external teams deployed Tiny Targets, a lack of information and communication, stereotypes applied by communities towards the deployment teams and the impression of inadequate respect for local customs led to anxiety and a misleading interpretation of the purpose of Tiny Targets and negatively influenced acceptability. This study highlights the importance of involving communities for programme acceptance. Our research underlined how awareness campaigns and communication are essential, but also how working within the scope of community social norms and customs are equally important. Prospects for the successful use of Tiny Targets are greater when communities are involved because the use can be adapted to social norms.

**Key questions**

**What is already know about this subject?**

- Community support and acceptability provides greater reassurance of vector control projects effectiveness and long-term success.

**What are the new findings?**

- Community acceptability was better when communities were actively involved in the vector control project.
- Research underlined how communication is essential and working within the scope of community social norms and customs are inescapable for community acceptability.

**What do the new findings imply?**

- Adequate time and effort must be invested in understanding, listening to and involving the people concerned before and during the implementation of vector control activities.

## Introduction

Human African trypanosomiasis (HAT), or sleeping sickness, is a fatal parasitic disease caused by *Trypanosoma brucei gambiense* or *T.b. rhodesiense,* both transmitted by tsetse flies. HAT is a disease unique to sub-Saharan Africa, where it affects mainly poor rural populations. Gambiense HAT (g-HAT) is an anthroponosis with a relatively slow progression whereas Rhodesiense HAT is an acutely progressing zoonotic disease.^1^ g-HAT accounted for >88% of all cases reported globally in 2019 (863/979) and 70% (604/863) of these occurred in the Democratic Republic of the Congo (DRC).^2^ In 2012, WHO presented a plan to eliminate HAT, first by reducing its incidence to very low levels by 2020 (<1 new case/year/10 000 people in 90% of known foci and <2000 cases/year reported globally) and, in a second stage, by interrupting transmission completely by 2030.^3 4^ Active case detection and treatment are the mainstays of efforts against g-HAT. This approach has led to a substantial decrease in the burden of g-HAT in DRC, with annual reported cases declining from 16 951 in 2000 to just 604 in 2019.^2^

Despite this reduction, epidemiological models predict that sole reliance on an active screening and treatment strategy will not interrupt transmission completely by 2030.^5^ The development of Tiny Targets, devices which attract and kill tsetse, offer a cost-effective means of controlling tsetse. The large scale of tsetse control operations make it difficult to conduct a classical cluster-randomised trial showing reduction in disease burden with implementation of the Tiny Targets intervention. Nonetheless, studies in Guinea,^6^ Chad^7^ and Uganda^8 9^ provide strong evidence that the use of Tiny Targets reduced the abundance of tsetse which, in turn, led to a lower incidence of gHAT.^10^

**Figure 1:**
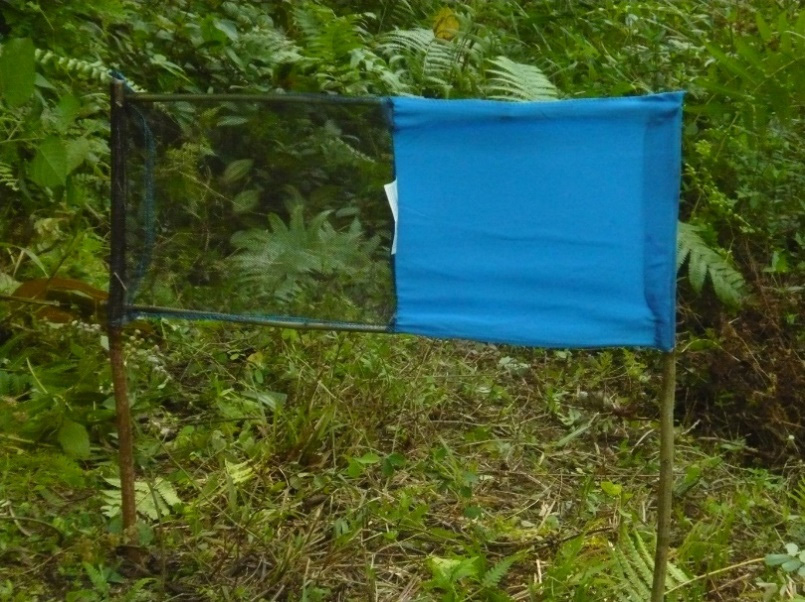
Tiny Target (credit: CVK).

Modelling studies also suggest that deployment of Tiny Targets will reduce incidence of gHAT.^11^ In persistent endemic areas, active screening may miss half of all cases^1^ resulting in continuing transmission.^12–15^ Mathematical models suggest that in these HAT foci the 2030 elimination goals could be achieved by adding vector control to screening and treatment.^5^ Tiny Targets, small (50×25 cm) panels of blue and black cloth impregnated with insecticide which attract (the blue part) and kill tsetse (black part, impregnated with insecticide), offer a proven, cost-effective vector control method (figure 1).^16 17^ Tiny Targets were first used experimentally in DRC in 2014, and were officially recognise as part of the HAT elimination strategy in 2019 by the DRC's Ministry of Health.

Previous studies on malaria and dengue have shown that successful vector control operations were associated with positive community reactions or behaviour towards the technology.^18–21^ Negative reactions were often linkedwith communities not being properly informed, consulted or involved. Several tsetse control projects in Uganda,^22 23^ Kenya,^24^ Ivory Coast,^25^ Ethiopia^24^ and Sudan^26^ have shownthat community acceptance is crucial for an intervention’s success. An example from the Republic of the Congo (Congo-Brazzaville) showed that misunderstanding and negative perceptions of the traps led to vandalism and destruction.^27–29^

Recently, two pilot vector control projects using Tiny Targets were implemented in three health zones in DRC. In 2014, a vector control project based on a strategy successfully employed in, Chad and Uganda was launched in DRC^1 6 30^ Tiny Targets were deployed by specialist intervention teams in this ‘programme-led’ (PL) strategy with limited involvement from surrounding communities. Then in 2017, a second project was introduced to test the feasibility of a community-based (CB) strategy in which Tiny Targets deployment was primarily managed and carried out by local community members. Both strategies proved to be successful, feasible and highly complementary^31 32^ and were both scheduled for scale-up commencing in 2019. However, prior to scale-up, community acceptability still needed to be explored as it provides greater reassurance of long-term success. Acceptability is the perception among individuals, organisations and entities involved in implementation that a given treatment, service, practice or innovation is agreeable or satisfactory.^33^

This paper compares and contrasts acceptability of Tiny Targets in two distinct village clusters: one in which Tiny Targets were deployed via a CB strategy and another in which Tiny Targets were deployed by a PL strategy.

## Methods

### Tiny Targets pilot projects implementation

The Tiny Target pilot projects were implemented in Kwilu province, one of the provinces in DRC most affected by HAT. Kwilu is east of Kinshasa province and apart from the capital Bandundu (470 km from Kinshasa); and the town of Kikwit (560 km from Kinshasa), the province is rural. Kwilu province is divided into 24 health zones, which are further divided into health areas.

**Figure 2:**
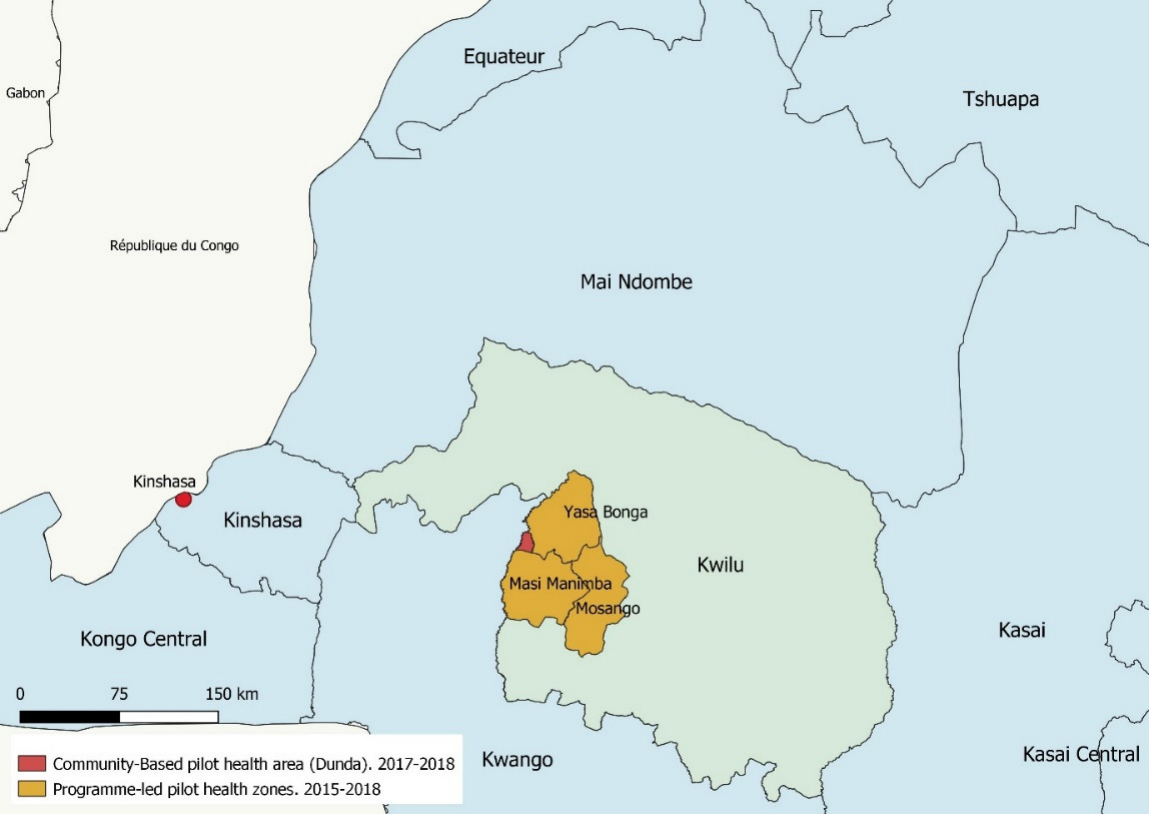
Pilot areas for vector control projects using Tiny Targets between 2015 and 2018, Kwilu Province, DRC. DRC, Democratic Republic of the Congo.

In 2015, the PL project was initiated progressively in three health zones (Yasa Bonga, Masi Manimba and Mosango).^32^ The CB strategy was implemented in three villages of Dunda health area part of Yasa Bonga health zone in 2017^31^ (figure 2).

The PL strategy was implemented by entomologists from the Liverpool School of Tropical Medicine (LSTM) together with counterparts from the Ministry of Health’s national sleeping sickness control programme, the Programme National de Lutte contre la Trypanosomiase Humaine Africaine (PNLTHA) in 2014. In the field, specialist teams recruited locally, trained and managed by LSTM and the programme, sporadically accompanied by a member of LSTM, travelled by boat along major rivers deploying Tiny Targets on the vegetated river banks where tsetse concentrate. Targets were re-deployed every 6 months. Surrounding communities of the targeted rivers are numerous but were not actively involved in the deployment due to logistical constraints and because most of the villages were located some distance from the river banks. Although a comprehensive sensitisation was planned this could not be fully executed. However radio messages from the health zone head doctor were broadcast at the beginning of the project to inform the population. Unfortunately, in those remote areas not everybody possesses a radio and the population were only partly informed. To try covering the sensitisation gap, when possible, the vector control team informed village chiefs and community members met during the implementation phase but visiting all the villages while deploying was not feasable.

Although the PL approach seemed to give satisfactory results, the simplicity of Tiny Targets makes them a strong candidate for a CB approach. In 2017, it was decided to test the feasibility of a Community-based vector control strategy targeting places where people get bitten. The CB strategy was managed by community members, with the support of a research team. The research team comprised three researchers, a Congolese anthropologist (RN), one doctor from PNLTHA (AM) and one anthropologist from the Institute of Tropical Medicine in Antwerp, Belgium (CVK). After obtaining agreement from the Health Zone authorities, the research team presented the Tiny Target project in first instance to the chief of each village, all chiefs were supportive. The research team then initiated a Tiny Target project in each village. They supported the creation of a vector control committee which offered training and support by providing information, dissemination of good practices along with technical and didactic material. They encouraged the committee to assume leadership of the project progressively, taking on organisation and management of the vector control activity. Vector control committees organised sensitisation campaigns in their respective communities and ensured the deployment of Tiny Targets. Community deployment further differed from the PL strategy in that Tiny Targets were not deployed along rivers but, instead, in and around fields, fishponds and places where community members had been bitten by tsetse.^31^

#### Timeframe, study area and population of the acceptability study

**Figure 3:**
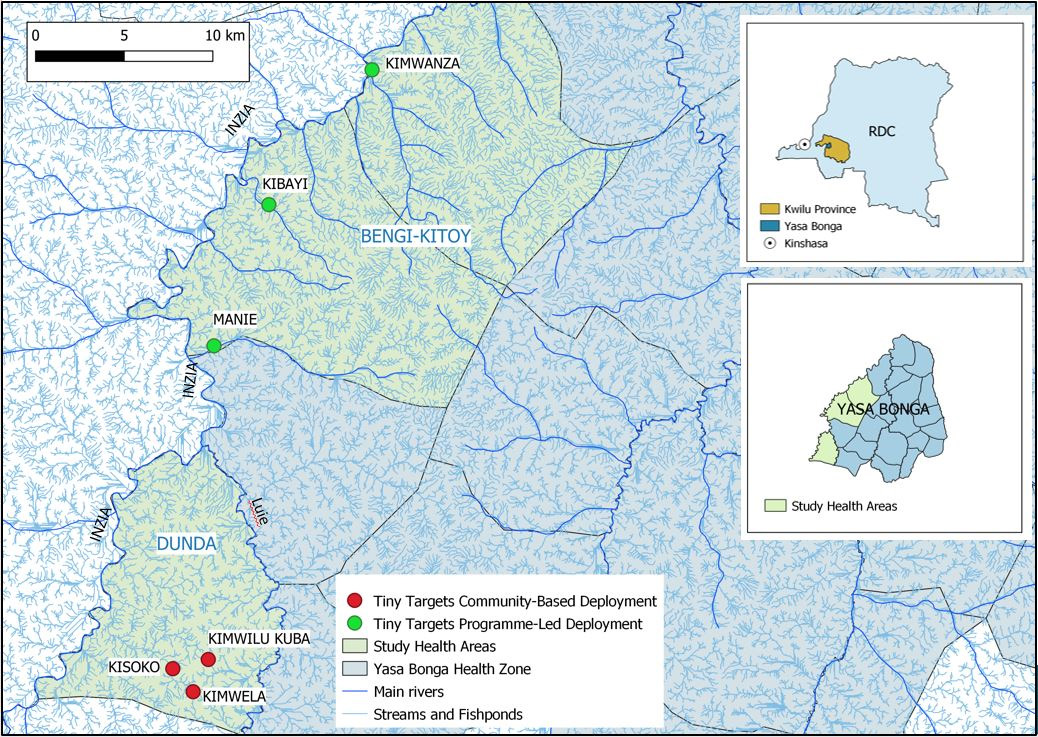
Villages of the study location, Yasa Bonga, Kwilu Province, Democratic Republic of Congo or RDC (Republique Démocratique du Congo (credit: CVK).

This study took place in February 2018, 6 months after the first deployment of Tiny Targets and before the planned scaling up, in six endemic villages. We selected the three pilot villages of the Dunda health area where the CB approach was implemented in August 2017: Kimwilu Kuba, Kimwela and Kisoko. Then we selected three villages near to where the PL team started the deployment also in 2017 and operated without the villagers’ involvement, 30–60 Km north of the Dunda (CB) villages, along the Inzia River in the Bengi and Kitoy health areas (Bengi-Kitoy): Kimwanza, Kibayi and Manie (figure 3).

Both areas have experienced active case detection for HAT by mobile screening units for decades and had a brief experience with a vector control using classical traps between 2008 and 2010.

The populations of the selected health areas are largely dependent on agriculture and fish farming. For the latter, ponds are created by sequentially damming tributaries of the rivers Luie and Inzia. This practice conserves natural riverine vegetation along the margins of the ponds and provides a favourable environment for tsetse.

In traditional Central African cultures, rivers are an important place for the Mamiwata figures, water spirits. The Bandundu population is rooted in the Kongo culture where there is a common belief in the existence of an invisible world having a strong influence on human daily lives, Mamiwata is part of this invisible world. They are often represented as mermaids, they are feared but also described as the incarnation of clan ancestors to protect villages.^34 35^ They are therefore very important and present in environments where water bodies are abundant.

### Data collection

Data were collected using two qualitative methods: focus group discussions (FGDs) and semistructured interviews (SSIs).

Fourteen FGDs were organised with an average of eight participants each. One male and one female (from 18 years old) FGD was convened per village alongside one mixed-gender FGD of young adults (18–30 years old). Members of the vector control committee in the Dunda (CB) health area were excluded from FGD participation as they were too involved with Tiny Target deployment to report on a broader villagers’ perception.

Seven SSIs with village traditional chiefs were also held (one of the villages is a fusion of two and has two chiefs) to obtain a community leaders’ perspective. All traditional chiefs were male, as few women hold this position in DRC. If traditional chiefs from the CB cluster were part of the vector control committee in the CB cluster, the interview was done with the assistant-chief.

FGDs and SSIs were held in a classroom outside school hours and lasted between 45 and 60 min. They were conducted in Kikongo by a Kikongo-speaking anthropologist (RN) supported by a local assistant. All FGDs and SSIs were recorded with participant permission. Table 1 shows the number of SSIs and FGDs held in each village.

**Table 1:** Composition of FGDs and SSIs for each village

FGDs and SSIs for each villages		FGD female	FGD male	FGD mixed	SSI chiefs
Dunda (CB)	Kimwilu Kuba	1	1	1	1
	Kimwela	1	1		1
	Kisoko	1	1		1
Bengi-Kitoy (PL)	Kibayi	1	1		1
	Kimwanza	1	1	1	1
	Manie	1	1		2
**Total**		**6**	**6**	**2**	**7**
**Total**		**14 FGDs**			**7 SSIs**

CB, community based; FGDs, focus group discussions; PL, programme led; SSIs, semistructured interviews.

### Data analysis

Audiorecorded FGDs and SSIs were translated from Kikongo into French and transcribed in a Word document, the translation was checked by a research team member fluent in both French and Kikongo (RN). The quotes reported in the paper were translated from French to English by the first author (CVK). All transcripts were cross-checked by two anthropologists (CVK and RN) to ensure accuracy and analysed using a thematic content analysis approach.^36^ This method combines a deductive approach, where data were analysed according to predefined themes, and then an inductive approach where novel themes were identified. All transcripts were carefully read and reread to recognise and identify patterns and compare similarities and differences between SSIs and FGDs or between FGDs categories and village clusters. NVivo software (V.11; QSR International, Melbourne, Australia) was used to support the data analysis. To increase internal validity a sociologist not involved in data collection (DP) reviewed the data and consistency of the coding system. Additionally, preliminary findings were shared and discussed with members of the broader research team, composed of professionals of diverse background such as epidemiology and vector control.

### Patient and public involvement

No patients or members of the public were involved in the design, conduct or reporting of this study.

## Results

Results are presented based on the predefined themes from the FGDs and SSIs: (1) Knowledge about the disease, (2) Perception of communication pathway, (3) Perception of Tiny Targets, (4) Perception of activity effectiveness and (5) Community recommendations for future activities. A marked difference was observed between village clusters. However, no differences were observed between gender and ages on topics discussed.

### Knowledge of sleeping sickness and tsetse flies: a favourable community background for Tiny Targets acceptance

Both village clusters were knowledgeable about HAT and tsetse. HAT was apparent in their collective consciousness; participants related many stories and experiences regarding sleeping sickness which they called Manimba (dozing) in Kikongo. They regarded screening and treatment activities in the region positively. They acknowledged that screening activities saved many lives and resolved many family conflicts in cases when disease symptoms were associated with witchcraft.

My son is still alive thanks to the nurses that screened and treated him. He was going crazy and we didn’t know what it was. We tested him and he had sleeping sickness. But in the past many family get separated (Due to conflicts) because some people thought someone put a spell on someone else, but it was in reality sleeping sickness. (FGD, Women, Bengi-Kitoy (PL), 2018)

Participants had a good knowledge of the disease, they all mentioned it was deadly and were aware of the neurological symptoms. The relatively low prevalence of sleeping sickness was recognised with participants reporting that nowadays there were fewer cases than in the past. For this reason, other diseases such as malaria were considered a greater health priority. Nevertheless, tsetse were widely considered a nuisance in daily life.

Tsetse are annoying insects in our village, it is a good thing that you come with this initiative to get rid of them, because they are really annoying, especially us the women who are going every day to the spings or cassava retting, it is really insecure (FGD, women, Bengi-Kitoy (PL), 2018)

Tsetse were recognised as the vector of sleeping sickness, although some participants believed it was also a malaria vector. Participants called tsetse kibisu, which referred to several insects with a painful bite; the black kibisu was specifically identified as a tsetse. Kibisu was described as the ‘enemy’ or ‘a sorcerer’.

### Perception of communication pathway

**Figure 4:**
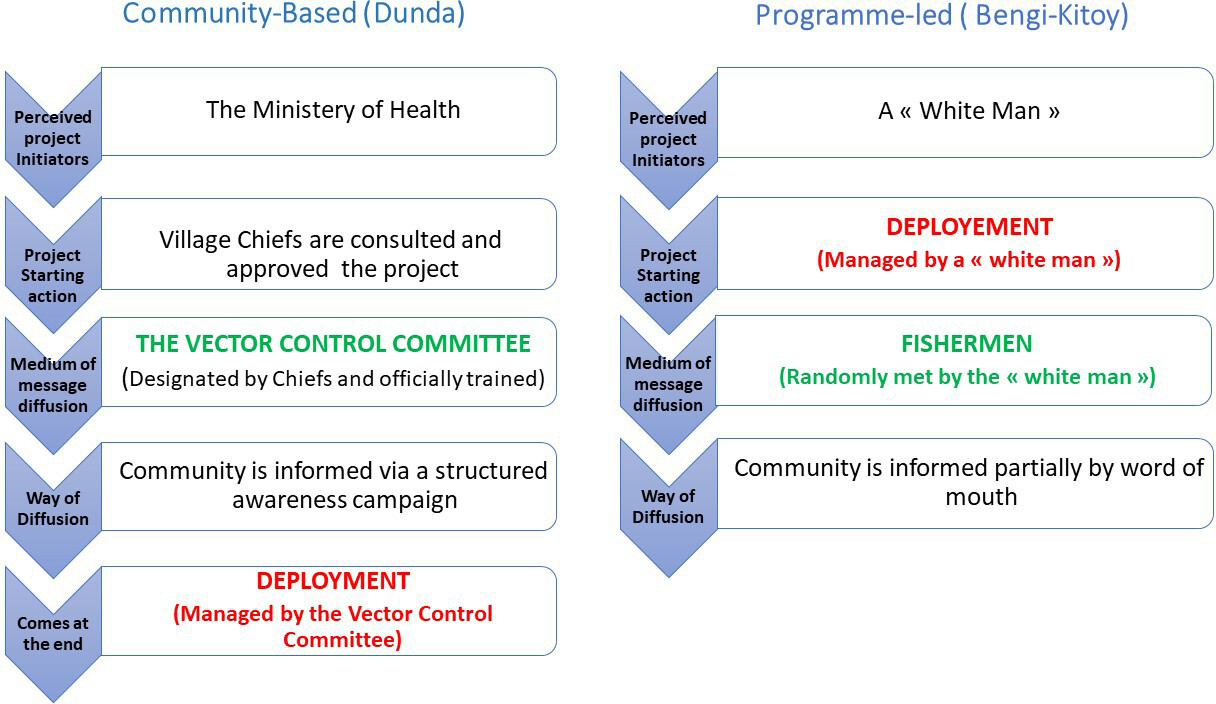
Village cluster’s perception of the deployment process and communication received on the process.

When participants of both Dunda (CB) and Bengi-Kitoy (PL) were asked how they received information about Tiny Targets and what they knew about them, we observed a marked difference. In Dunda (CB), participants described being informed prior to deployment and respecting community norms. In Bengi-Kitoy (PL) participants reported being informed partially after the deployment took place. Figure 4 summarises the differences in the communication pathway as described by community members.

In Bengi-Kitoy (PL), participants knew Tiny Targets were associated with sleeping sickness but had not received detailed information. Participants described the information flow as first a ‘white man’ coming and randomly talking with fishermen along the river. Then the fishermen communicated the information to some people in the rest of the community. This communication was described as unstructured, slowly and partially disseminated throughout the community by word of mouth. Therefore, the information received was not always perceived as legitimate.

Fishermen know more about the Tiny Targets, because this “white man” gave them information…and then fishermen gave us an explanation. But they explain things they don’t really know. (FGD, Bengi-Kitoy (PL), Men, 2018)

In Dunda (CB), in contrast, participants knew about the use and purpose of Tiny Targets in detail. They described that the information provided was reliable and organised in a structured way by the vector control committee. They reported that at the beginning they believed the project was initiated by the ‘white woman’ but finally they were explained it was a project initiated by the Ministry of Health.

Both village clusters stressed that endorsement by the chief was a very important aspect for good communication and acceptability. In Dunda (CB), the committee was perceived as legitimate because the chiefs supported the activity and organised the committee members selection.

The chief accepted the project and he informed the population that it was to implement a project against the tsetse flies in our village. Those who wanted to participate expressed their interest and constituted the committee. They were trained and they are doing a good job. People accept the project. (FGD, Young, Dunda, 2018)

In Bengi-Kitoy (PL), participants and more particularly chiefs expressed their dissatisfaction and frustration regarding the vector control team’s attitude and lack of respect for the authorities.

You know, when you arrive to someone, you start with a knock on the door, if we open you enter, otherwise you stay out. This is the same situation: The first thing you do is go to the chief to tell him what you are coming for, then the chief can inform the population. We also want to terminate the disease, we would like to collaborate, but this “white man” he is just passing. (FGD, Bengi-Kitoy (PL), Women, 2018)

However, Chiefs from Bengi-Kitoy (PL) reported that they will respect the Tiny Targets because they heard a radio message by the health zone head doctor, perceived as a higher authority, asking people not to destroy the Tiny Targets.

We cannot take them out, some doctor and nurses from the health centre sent us messages to ask not to remove them. So we don’t want to have problems with authorities. Then we also thought it was probably for our health. So nobody is taking them out (SSI, Bengi-Kitoy (PL), Chef 2, 2018)

### Perception of Tiny Targets: consequences of the communication pathways

The differences of the communication pathways and the respect, or lack thereof, for community norms had a clear influence on perception of Tiny Targets. A marked difference between the acceptability level of Dunda (CB) and Bengi-Kitoy (PL) was observed. In Dunda (CB), despite some initial doubts about the use of the Tiny Targets, people accepted the project relatively rapidly.

It was the first time we saw Tiny Targets, we didn’t know about them. At first we didn’t trust the Tiny Targets. It is only after receiving information from the committee that we learnt it was to protect us from sleeping sickness. We were happy then. When they started deploying Tiny Targets around the ponds, everybody wanted one near theirs (FGD, Dunda (CB), Men, 2018)

In Bengi-Kitoy (PL) there was much more confusion. Participants all reported that at the beginning, acceptability was limited and the acceptance process went slowly. Although they said they finally accepted them, they also frequently mentioned the fact that still today many rumours are circulating and they still have some doubts about the purpose of the Tiny Targets.

Before putting the Tiny Targets up, it is necessary to inform the population because it concerns us and lack of information leads to fear and rumours. Some people understand it is for sleeping sickness but many are telling a lot of rumors (FGD, Bengi-Kitoy (PL), Women 2018)

In Bengi-Kitoy (PL) there were suspicions over the intended purpose of the tool itself. People remembered the previous method using traps that caught flies and they were confused about Tiny Targets that did not catch but only kill flies; and concluded the intention was not to catch flies but something else.

People ask themselves where are the dead flies? Before, (NDRL in 2008 2010) we brought the flies to nurse Doris in Kitoy. This is why there is people who think this “white man” came to look for something in the water, but there is also people who says it is for the tsetse flies (FGD, Bengi-Kitoy (PL), Women, 2018)

Then there were suspicions over the deployment strategy. People did not understand why Tiny Targets were only deployed along the Inzia River as most of the community members were working away from the river in fields or ponds. This was not the case in Dunda (CB) where the community was deploying the Tiny Targets around ponds or in fields.

People said he (the ‘white man’) came to look for something in the water because he is only putting the Tiny Target along the river. If he was putting them everywhere people won’t say that. He has to put the Tiny Target everywhere to prove us Tiny Target are there for our protection. Tsetse flies are not only in the river Inzia. (FGD, Bengi-Kitoy (PL), Women, 2018)

There was suspicion or fear regarding this unknown ‘white man’, whose attitude was perceived as avoiding contact with the community.

…The population is asking if he (‘white man’) is really coming for helping us, because he is not explaining anything, he is not talking with us, so people think he may be here for something else. (FGD, Bengi-Kitoy (PL), Men, 2018)

Participants reported that all those suspicions led many people to perceive a possible danger for the community that created fear and anxiety.

Since he put the Tiny Targets up, there are a lot of women who don’t go into the forest anymore because they are scared the ‘white man’ came to kill us… (FGD, Bengi-Kitoy (PL), Women, 2018)

The most common possible risk perceived by the participants of Bengi-Kitoy (PL) was the ‘white man’ may have witchcraft intentions and was there to steal the Mamiwata.

There is a lot of wealth in our rivers. There are spirits left by ancestors in the water to protect the village. White men like the Mamiwata, we know that. They can come to take them and bring them home. It is a lot of money. (FGD, Bengi-Kitoy (PL), Woman, 2018)

In Dunda (CB) possible risks caused by Tiny Targets were also reported. At the beginning a few participants reported they were worried about the intentions of the ‘white woman’ and the stealing of Mamiwata. However, the main perceived risks were rather linked with the possible effect of the insecticide present on Tiny Targets on soil impoverishment. While erroneous, this concern seemed related to a poor harvest of peanuts in 2017 which reinforced these rumours and some people refused to have the Tiny Targets too close to their fields or ponds.

Some people said the Tiny Targets impoverished the soil and caused the weak peanuts harvest. However, committee members told them that it is not the first time we have a weak harvest and it is in every village, not only the ones who deployed Tiny Targets. Finally they understood. (FDG, Dunda (CB), Women, 201

### Perception of the Tiny Targets effectiveness

Tiny Targets were perceived as very effective in the Dunda (CB) cluster. In all FGDs or interviews, participants perceived significant reductions in biting by tsetse.

There is a big change because since there have been Tiny Targets we don’t see tsetse flies anymore. Tsetse doesn’t bite people in the forest like before. (FGD, Women, Dunda (CB), 2018)

However, some participants mentioned the programme was not effective enough because more Tiny Targets were needed. Others reported community members were suspicious because they could not see dead tsetse flies.

In Bengi-Kitoy (PL), the perception of effectiveness was more varied. Some participants observed a decrease in biting rates, while others expressed suspicions and doubts about the efficacy of the deployment strategy. As mentioned above, they did not understand why Tiny Targets were only deployed along the Inzia main river and not in other places like the fishponds where people got bitten, they also reported not perceiving diminution of bites. They felt that fishermen were therefore privileged by the deployment strategy.

I m a fishermen, when this ‘white men’ is coming at the river we work with him, but I think he is not doing right, he is putting Tiny Targets at the river but tsetse flies are everywhere. He should also put them at our springs near the village. This is why until now tsetse flies still resist where there are no Tiny Targets (FGD, Bengi-Kitoy (PL), Men, 2018

Participants from Bengi-Kitoy (PL) expressed doubts about the effectiveness of Tiny Targets as a vector control tool, sceptical about the fact that Tiny Targets did not catch flies.

If you want to eradicate tsetse flies you need to provide us the old model [tsetse fly traps] because the one in the river does not catch flies, they are not good. (FGD, Men, Bengi-Kitoy (PL), 2018)

### Community recommendations for future activities: importance of being involved

In the Dunda (CB) cluster, the current situation was globally described as satisfactory and no major comments were made when asking participants for future advice. They expressed satisfaction with the committee’s work, that they all help the committee in reporting when Tiny Targets need maintenance, they wanted the project to continue and hoped the research team would not abandon them.

Our wish is the project being permanent in order to eliminate the sleeping sickness in our village. Be with us until the sleeping sickness disappears from our village. Continue to bring Traps and Tiny Targets to get rid of tsetse flies (FGD, Men, Dunda (CB), 2018)

In Bengi-Kitoy (PL), participants also said they wanted the project to continue but strongly expressed that they felt left out and that they wanted to participate. The lack of involvement created a certain anxiety and suspicion.

This ‘white men’ came to protect us against tsetse flies, so why he does not involve us?… He is putting the tiny targets like he knows better our forest (FGD, Men, Bengi-Kitoy (PL), 2018)

Another source of anxiety reported by some participants was the lack of control about their environment. Some mentioned existing taboos and worries about transgressing them if the population is not consulted.

There is some spirits that we can see during the night, because they go out at night, they bathe at night. They can be very scary, if someone goes there at night… Us, the fishermen, we know those places where spirits are and where we have to be very careful not to disturb them. They protect the innocents, but if you do something bad to them they won’t protect you anymore. (FGD, Men, Bengi-Kitoy (PL), 2018)

Finally, participants wanted to get involved simply because they thought it would make the project more effective by covering more accurate places, where people got bitten.

This white has to ask us where to put the Tiny Targets if he wants to get rid of Tsetse Flies. They are not only at the river. (FGS, Men, Bengi-Kitoy (PL), 2018

Table 2 summarises all the Tiny Targets acceptability factors highlighted on the FGDs and SSIs conducted in PL and CB clusters.

**Table 2:** Summary of the different Tiny Targets acceptability elements reported by participants in Dunda (CB) and Bengi-Kitoy (PL), 2018

Acceptability elements	Reported by dunda (CB)	Reported by Bengi-Kitoy (PL)
Previous knowledge about the disease	**+**	**+**
Perception importance of the disease	−	−
Perception of nuisances caused by vector	**+**	**+**
Perception of good information received	**+**	−
Perception of Tiny Targets efficacy	**+**	**+/−**
Perception of TIny Targets placement strategy effectiveness	**+**	−
Perception of no negative side effects (for health or environment)	**+/−**	Not mentioned
No supernatural danger perceived	**+/−**	−
No anxiety caused by community outsiders	**+/−**	−
Assuming control feeling	Not mentioned	−

+, present, −, not present,+/−, partly present.

CB, community based; PL, programme led.

## Discussion

Our findings show, that in villages where the community led vector control activities, people had a positive perception towards Tiny Targets. Conversely, where community members were less involved, acceptability was reduced.

This study highlights, in line with others, that acceptability of a new vector control technology is a result of a combination of several important factors including: prior knowledge of the disease, perception of the vector’s nuisance, effective raising of awareness, perceived effectiveness of the technology and absence of side effects.^18–21 31 37–47^

Our findings from Bengi-Kitoy (PL) suggested that previous knowledge of the disease and perception of vector nuisances was not sufficient to understand the link between the newly introduced Tiny Target and sleeping sickness. Therefore, as with previous studies, our research shows that improving awareness about the function and benefits of vector control tools is a crucial element for acceptability.^31 40 42 45^ In Bengi-Kitoy (PL), where information was scarce, the presence of Tiny Targets raised many unanswered questions leading to suspicions and anxiety. This anxiety is even more understandable given that water bodies are closely linked with supernatural power and ancestors’ legacies which are perceived as having a strong influence on the stability of daily life.^29 34 35 45 46^ Therefore, our study also suggests that ‘no vandalism’ may be an indicator of fear and it may be misleading to see it as an indicator of acceptance. Similar to Kovacic’s study^45^ on acceptability of tsetse traps, respondents reported not vandalising Tiny Targets because of a perceived danger that they might be linked to witchcraft and kill them. In contrast, in Dunda (CB) where the information was well disseminated, most people perceived the Tiny Targets as beneficial and links made to witchcraft were anecdotal.

Awareness raising and communication has to be introduced at the start of any vector control activity but also to be continuous to respond to doubts and questions emerging during the process. For instance, a study in Tanzania on acceptability of attractive toxic sugar baits to control mosquitoes reported that people regarded the strategy as being ineffective even if they had received good initial information and noticed a reduction in mosquito nuisance. The tool was used outdoors whereas people perceived malaria transmission to be higher indoors.^40^ In our study, the village cluster involved in the CB approach did not question the deployment strategy because community members placed Tiny Targets where they perceived the risk of being bitten to be the highest. In contrast, in the other cluster, they did not perceive Tiny Targets as beneficial as they only noted a reduction in nuisance at the river banks where risk is not perceived to be high and they did not understand the deployment logic. Hence, it is essential to provide the community with the necessary technical knowledge to address doubts about the process.

Although continuous awareness raising and maintaining dialogue are essential to ensure good understanding of the Tiny Target tool, the deployment strategy and the benefits, they are not sufficient to guarantee acceptability. Our study particularly highlights the value of implementing projects within the sphere of local customs. For instance, participants expressed the importance of respecting sacred places and taboos while deploying Tiny Targets, and the endorsement by recognised local authorities for the project to be trusted. A study in Mozambique showed that indoor residual spraying for malaria was not perceived as being particularly effective but as it was implemented and supported by the trusted authorities acceptability was high.^47^ In Dunda (CB), communities appreciated the fact their traditional chiefs endorsed and were involved in the project. In contrast, Bengi-Kitoy (PL) communities deplored the fact that their chiefs were not informed which added to their suspicions about the intervention.

Finally, our findings underlined how images, representations and existing stereotypes based on skin colour, ethnicity and related Congolese history highly influence acceptability in our study context. The ‘white man’ was frequently mentioned in all FGDs and interviews from the Bengi-Kitoy (PL) cluster and appeared to be a central preoccupation. The technical support of ‘white’ entomologists to the national programme local deployment team was in reality sporadic. A majority of participants had not actually seen the ‘white man’ in the region, only hearing about him from fishermen. However, stereotypes that Congolese rural communities have regarding ‘white people’ plus their rarity in such remote places made this event central, distorting the perception of reality and exacerbating rumours created by the lack of information. In Dunda (CB), the project managers were identified as being part of the community, people were informed about the project as well as about the role of the ‘white’ anthropologist. Although people were suspicious about the anthropologist, those suspicions were not reported as being a central preoccupation or a source of anxiety. This study showed particularly that acceptability is influenced by stereotypes that communities may have about those they identified as project planners and the way these planners are managing their activities and respecting the local customs.

We recommend that vector control planners external to communities invest the time and effort to open a dialogue with communities. This dialogue provides a means to identify important acceptability factors and, more importantly, to identify opportunities for community involvement. When people are involved, they have more opportunities to express how they want things to be done, retain control of culturally sensitive issues and respect for their norms and customs that outsiders cannot comprehend. The less communities are involved, the more likely the project initiatives are to displease or even shock people and be a serious barrier to project acceptability.

### Limitations

This study was conducted in two different village clusters. One cluster (Dunda (CB)) was composed of three villages that had already been part of a CB project for over 1 year. FGD participants from this cluster knew the research team and the vector control committee members from their villages and, therefore, may have been more reluctant to report criticism of the Tiny Target programme as compared with participants from the other cluster. This study was conducted at the end of the pilot implementation of the PL strategy and did not help to improve acceptability at earlier stages. It is advisable to conduct acceptability studies prospectively and in parallel with evaluations of effectiveness. The results of this study, as for other acceptability studies, should be interpreted with caution because acceptability varies depending on the sociocultural context. Finally, the approach adopted is cross-sectional and acceptability might change over time.

## Conclusion

This study is an additional example of the importance of involving communities for programme acceptance. Research underlined how awareness campaigns and communication are essential, but also how working within the scope of community social norms and customs are inescapable. Acceptability factors are numerous, complex and vary according to local factors. Adequate time and effort must be invested in understanding, listening to and involving the people concerned before and during the implementation of vector control activities.

## Data Availability

Data may be obtained from a third party and are not publicly available. The data supporting the findings of this study/publication are retained at the Institute of Tropical Medicine, Antwerp and will not be made openly accessible due to ethical and privacy concerns. Data can, however, be made available after approval of a motivated and written request to the Institute of Tropical Medicine at ITMresearchdataaccess@itg.be.

## References

[1] Tirados I, Esterhuizen J, Kovacic V, et al. Tsetse control and Gambian sleeping sickness; implications for control strategy. PLoS Negl Trop Dis 2015;9:e0003822. 10.1371/journal.pntd.0003822 26267814 PMC4580652

[2] World Health Organisation. Global health Observatory data Repository, number of new reported cases (T.b. gambiense) data by country. Available: http://apps.who.int/gho/data/node.main.A1636?lang=en2019http://apps.who.int/gho/data/node.main.A1636?lang=en

[3] Simarro PP, Cecchi G, Franco JR, et al. Monitoring the progress towards the elimination of gambiense human African trypanosomiasis. PLoS Negl Trop Dis 2015;9:e0003785. 10.1371/journal.pntd.0003785 26056823 PMC4461311

[4] Holmes P. First WHO meeting of stakeholders on elimination of gambiense human African trypanosomiasis. PLoS Negl Trop Dis 2014;8:e3244. 10.1371/journal.pntd.0003244 25340404 PMC4207655

[5] Solano P, Torr SJ, Lehane MJ. Is vector control needed to eliminate gambiense human African trypanosomiasis? Front Cell Infect Microbiol 2013;3:33. 10.3389/fcimb.2013.00033 23914350 PMC3728477

[6] Courtin F, Camara M, Rayaisse J-B, et al. Reducing Human-Tsetse contact significantly enhances the efficacy of sleeping sickness active screening campaigns: a promising result in the context of elimination. PLoS Negl Trop Dis 2015;9:e0003727. 10.1371/journal.pntd.0003727 26267667 PMC4534387

[7] Mahamat MH, Peka M, Rayaisse J-B, et al. Adding tsetse control to medical activities contributes to decreasing transmission of sleeping sickness in the Mandoul focus (Chad). PLoS Negl Trop Dis 2017;11:e0005792. 10.1371/journal.pntd.0005792 28750007 PMC5549763

[8] Bessell PR, Esterhuizen J, Lehane MJ, et al. Estimating the impact of tiny targets in reducing the incidence of Gambian sleeping sickness in the north-west Uganda focus. Parasit Vectors 2021;14:999–1000. 10.1186/s13071-021-04889-x PMC8371857 34407867

[9] Tirados I, Esterhuizen J, Kovacic V, et al. Tsetse control and Gambian sleeping sickness; implications for control strategy. PLoS Negl Trop Dis 2015;9:e0003822. 10.1371/journal.pntd.0003822 26267814 PMC4580652

[10] Tirados I, Hope A, Selby R, et al. Impact of tiny targets on Glossina fuscipes quanzensis, the primary vector of human African trypanosomiasis in the Democratic Republic of the Congo. PLoS Negl Trop Dis 2020;14:e0008270. 10.1371/journal.pntd.0008270 33064783 PMC7608941

[11] Rock KS, Torr SJ, Lumbala C, et al. Predicting the impact of intervention strategies for sleeping sickness in two High-Endemicity health zones of the Democratic Republic of Congo. PLoS Negl Trop Dis 2017;11:e0005162. 10.1371/journal.pntd.0005162 28056016 PMC5215767

[12] Jamonneau V, Ilboudo H, Kaboré J, et al. Untreated human infections by Trypanosoma brucei gambiense are not 100% fatal. PLoS Negl Trop Dis 2012;6:e1691. 10.1371/journal.pntd.0001691 22720107 PMC3373650

[13] Bucheton B, MacLeod A, Jamonneau V. Human host determinants influencing the outcome of Trypanosoma brucei gambiense infections. Parasite Immunol 2011;33:438–47. 10.1111/j.1365-3024.2011.01287.x 21385185 PMC3427891

[14] Mulenga P, Chenge F, Boelaert M, et al. Integration of human African trypanosomiasis control activities into primary healthcare services: a scoping review. Am J Trop Med Hyg 2019;101:1114–25. 10.4269/ajtmh.19-0232 31482788 PMC6838596

[15] Mpanya A, Hendrickx D, Vuna M, et al. Should I get screened for sleeping sickness? A qualitative study in Kasai Province, Democratic Republic of Congo. PLoS Negl Trop Dis 2012;6:e1467. 10.1371/journal.pntd.0001467 22272367 PMC3260312

[16] Rayaisse JB, Esterhuizen J, Tirados I, et al. Towards an optimal design of target for tsetse control: comparisons of novel targets for the control of Palpalis group tsetse in West Africa. PLoS Negl Trop Dis 2011;5:e1332. 10.1371/journal.pntd.0001332 21949896 PMC3176748

[17] Esterhuizen J, Rayaisse JB, Tirados I, et al. Improving the cost-effectiveness of visual devices for the control of riverine tsetse flies, the major vectors of human African trypanosomiasis. PLoS Negl Trop Dis 2011;5:e1257. 10.1371/journal.pntd.0001257 21829743 PMC3149014

[18] Kuadima JJ, Timinao L, Naidi L, et al. Long-term acceptability, durability and bio-efficacy of ZeroVector^®^ durable lining for vector control in Papua New Guinea. Malar J 2017;16:93. 10.1186/s12936-017-1742-y 28241875 PMC5329951

[19] Vanlerberghe V, Villegas E, Jirarojwatana S, et al. Determinants of uptake, short-term and continued use of insecticide-treated curtains and jar covers for dengue control. Trop Med Int Health 2011;16:162–73. 10.1111/j.1365-3156.2010.02668.x 21044236

[20] Pérez D, Van der Stuyft P, Toledo ME, et al. Insecticide treated curtains and residual insecticide treatment to control Aedes aegypti: an acceptability study in Santiago de Cuba. PLoS Negl Trop Dis 2018;12:e0006115. 10.1371/journal.pntd.0006115 29293501 PMC5766245

[21] Liverani M, Charlwood JD, Lawford H, et al. Field assessment of a novel spatial repellent for malaria control: a feasibility and acceptability study in Mondulkiri, Cambodia. Malar J 2017;16:412. 10.1186/s12936-017-2059-6 29029614 PMC5640900

[22] Kovacic V. Women-led tsetse control: a pilot study in northwest Uganda. University of Liverpool, 2015.

[23] Okoth JO, Kirumira EK, Kapaata R. A new approach to community participation in tsetse control in the Busoga sleeping sickness focus, Uganda. A preliminary report. Ann Trop Med Parasitol 1991;85:315–22. 10.1080/00034983.1991.11812567 1746980

[24] Barrett K, Okoli C. Community participation in the management of tsetse. A comparative assessment of impact and sustainability. DFID 1998.

[25] Laveissiere C, Hervouet J, Couret D, et al. La campagne pilote de lutte contre la trypanosomiase humaine dans le foyer de Vavoua (Côte d'Ivoire). Cah ORSTOM, sér, ENtméd et Parasitol 1985;XXIII:167–85.

[26] Joja LL, Okoli UA. Trapping the vector: community action to curb sleeping sickness in southern Sudan. Am J Public Health 2001;91:1583–5. 10.2105/ajph.91.10.1583 11574312 PMC1446831

[27] Gouteux JP, Bansimba P, Bissadidi N, et al. [Responsibility for tsetse control by rural communities: first trial in 5 Congolese villages]. Ann Soc Belg Med Trop 1987;67:37–49. 3632076

[28] Gouteux JP, Sinda D. Community participation in the control of tsetse flies. Large scale trials using the pyramid trap in the Congo. Trop Med Parasitol 1990;41:49–55. 2339247

[29] Leygues M, Gouteux JP. [A community battle against a tropical endemic disease: supernatural beliefs and tsetse fly traps in the Congo]. Soc Sci Med 1989;28:1255–67. 10.1016/0277-9536(89)90344-4 2734626

[30] Mahamat MH, Peka M, Rayaisse J-B, et al. Adding tsetse control to medical activities contributes to decreasing transmission of sleeping sickness in the Mandoul focus (Chad). PLoS Negl Trop Dis 2017;11:e0005792. 10.1371/journal.pntd.0005792 28750007 PMC5549763

[31] Vander Kelen C, Mpanya A, Boelaert M, et al. Feasibility of community-based control of tsetse: a pilot project using tiny targets in the Democratic Republic of Congo 2020. 10.1371/journal.pntd.0008696 PMC7537905 32970689

[32] Tirados I, Hope A, Selby R, et al. Impact of tiny targets on Glossina fuscipes quanzensis, the primary vector of human African trypanosomiasis in the Democratic Republic of the Congo. PLoS Negl Trop Dis 2020;14:e0008270. 10.1371/journal.pntd.0008270 33064783 PMC7608941

[33] Proctor E, Silmere H, Raghavan R, et al. Outcomes for implementation research: conceptual distinctions, measurement challenges, and research agenda. Adm Policy Ment Health 2011;38:65–76. 10.1007/s10488-010-0319-7 20957426 PMC3068522

[34] Hagenbucher Sacripanti F. Santé et rédemption par les génies au Congo. Publisud, 1989.

[35] Hagenbucher Sacripanti F. La représentation culturelle traditionelle de la Trypanosomiase dans le Niari (République Populaire du Congo). Cah ORSTOM, sér Sci Hum 1981-1982;XVIII:445–73.

[36] Green J, Thorogood N. Qualitative mthods for health research. 2004. 3rd ed. London: Sage, 2014.

[37] Sekhon M, Cartwright M, Francis JJ. Acceptability of healthcare interventions: an overview of reviews and development of a theoretical framework. BMC Health Serv Res 2017;17:88. 10.1186/s12913-017-2031-8 28126032 PMC5267473

[38] Dambach P, Jorge MM, Traoré I, et al. A qualitative study of community perception and acceptance of biological larviciding for malaria mosquito control in rural Burkina Faso. BMC Public Health 2018;18:399. 10.1186/s12889-018-5299-7 29566754 PMC5865284

[39] Pulford J, Tandrapah A, Atkinson J-A, et al. Feasibility and acceptability of insecticide-treated plastic sheeting (ITPS) for vector control in Papua New Guinea. Malar J 2012;11:342. 10.1186/1475-2875-11-342 23046535 PMC3507715

[40] Maia MF, Tenywa FC, Nelson H, et al. Attractive toxic sugar baits for controlling mosquitoes: a qualitative study in Bagamoyo, Tanzania. Malar J 2018;17:22. 10.1186/s12936-018-2171-2 29321011 PMC5763615

[41] Kamuanga M, Sigué H, Swallow B, et al. Farmers' perceptions of the impacts of tsetse and trypanosomosis control on livestock production: evidence from southern Burkina Faso. Trop Anim Health Prod 2001;33:141–53. 10.1023/A:1005286631470 11254074

[42] Shafique M, Lopes S, Doum D, et al. Implementation of guppy fish (Poecilia reticulata), and a novel larvicide (Pyriproxyfen) product (Sumilarv 2MR) for dengue control in Cambodia: a qualitative study of acceptability, sustainability and community engagement. PLoS Negl Trop Dis 2019;13:e0007907. 10.1371/journal.pntd.0007907 31738759 PMC6886868

[43] Makungu C, Stephen S, Kumburu S, et al. Informing new or improved vector control tools for reducing the malaria burden in Tanzania: a qualitative exploration of perceptions of mosquitoes and methods for their control among the residents of Dar es Salaam. Malar J 2017;16:410. 10.1186/s12936-017-2056-9 29020970 PMC5637339

[44] Messenger LA, Rowland M. Insecticide-treated durable wall lining (ITWL): future prospects for control of malaria and other vector-borne diseases. Malar J 2017;16:213. 10.1186/s12936-017-1867-z 28532494 PMC5441104

[45] Kovacic V, Tirados I, Esterhuizen J, et al. Community acceptance of tsetse control baits: a qualitative study in Arua District, North West Uganda. PLoS Negl Trop Dis 2013;7:e2579. 10.1371/journal.pntd.0002579 24349593 PMC3861179

[46] Gouteux JP. [The supernatural, health and community action in Black Africa]. Bull Soc Pathol Exot 1992;85:256–60. 1422280

[47] Montgomery CM, Munguambe K, Pool R. Group-based citizenship in the acceptance of indoor residual spraying (IRS) for malaria control in Mozambique. Soc Sci Med 2010;70:1648–55. 10.1016/j.socscimed.2010.01.020 20199837

